# Mechanical Stretch-Induced NLRP3 Inflammasome Expression on Human Annulus Fibrosus Cells Modulated by Endoplasmic Reticulum Stress

**DOI:** 10.3390/ijms23147951

**Published:** 2022-07-19

**Authors:** Hsin-I Chang, Cheng-Nan Chen, Kuo-Yuan Huang

**Affiliations:** 1 Department of Biochemical Science and Technology, National Chiayi University, Chiayi 60004, Taiwan; hchang@mail.ncyu.edu.tw (H.-I.C.); cnchen@mail.ncyu.edu.tw (C.-N.C.); 2 Department of Orthopedics, National Cheng Kung University Hospital, College of Medicine, National Cheng Kung University, Tainan 70403, Taiwan

**Keywords:** annulus fibrosus, mechanical stretch, endoplasmic reticulum stress, NLRP3 inflammasome, reactive oxygen species, intervertebral disc degeneration

## Abstract

Excessive mechanical loading is a major cause of spinal degeneration, typically originating from a tear in the annulus fibrosus (AF). Endoplasmic reticulum (ER) stress and NLRP3 (NOD-, LRR- and pyrin domain-containing protein 3) inflammasome have been implicated in the pathogenesis of intervertebral disc (IVD) degeneration. However, the causal relationship between the mechanical stretching of AF cells and the NLRP3 inflammasome response associated with ER stress remains scarce. To elucidate the pathogenesis and regulatory mechanisms of mechanical stretch-induced IVD degeneration, human AF cell lines were subjected to different degrees of cyclic stretching to simulate daily spinal movements. Our results indicated that 15% high cyclic stretch (HCS) induced the expression of NLRP3 and interleukin-1 beta (IL-1β) and was also responsible for the increased expression of NADPH (nicotinamide adenine dinucleotide phosphate) oxidase 2 (NOX2) and reactive oxygen species (ROS) in human AF cells. In addition, HCS increased the expression of glucose-regulated protein 78 (GRP78), an ER stress chaperone, which was neutralized with tauroursodeoxycholic acid (TUDCA), an ER stress inhibitor. In addition, HCS was found to induce thioredoxin-interacting protein (TXNIP) expression and NLRP3 inflammasome activation, which can be suppressed by si-NOX2 or the NOX2 inhibitor GSK2795039. Consequently, HCS upregulated ER stress and ROS production, leading to increased NLRP3 and IL-1β expression in human AF cells, and may further accelerate IVD degeneration.

## 1. Introduction

Spinal degeneration usually originates from intervertebral disc (IVD) injury [1]. Degenerative disc disease (DDD) is the leading cause of discogenic low back pain [1] and can also become an enormous socio-economic burden and influence an individual’s quality of life [2]. The etiologies of DDD include repetitive cyclic stress, aging, inflammation, nutrition, genetic predisposition, etc. [3,4,5,6,7,8,9,10]. Excessive mechanical loading is a potential cause of IVD degeneration [6]. The IVD consists of three main structures, including the inner nucleus pulposus (NP), outer annulus fibrosus (AF), and superior and inferior cartilaginous endplates, which provide flexibility to the spine and help resist mechanical stress [11]. Repetitive cyclic stress from daily movements of the spine can initiate AF damage, and this may lead to IVD degeneration [12].

The endoplasmic reticulum (ER) is an important subcellular organelle that regulates intracellular calcium homeostasis, protein synthesis and apoptosis [13]. ER stress signaling pathways, collectively known as the unfolded protein response (UPR), serve as a quality control mechanism to restore ER homeostasis and function under the conditions of stress. However, when the stress is extreme or persistent, the resulting dysfunction of the ER may compromise the cells, leading to apoptosis [14]. ER stress can not only affect cell survival or death through autophagy, but also directly induce cell death [15]. ER stress is associated with a variety of degenerative diseases, such as Parkinson’s disease, Alzheimer’s disease, chronic osteoarthritis, etc. [16,17,18]. In addition, ER stress has been shown to be associated with the progression of IVD degeneration [19]. For example, ER stress induced by certain pathological factors tends to promote the apoptosis of nucleus pulposus (NP) cells [19,20,21]. Previous studies have demonstrated that high cyclic stretch (HCS) could induce reactive oxygen species (ROS) production, the expression of ER stress markers (e.g., glucose-regulated protein 78 (GRP78)), and cell apoptosis in AF cells, respectively [13,22,23]. However, the mechanism by which HCS regulates the NLRP3 inflammasome-associated ER stress response has not been reported in AF cells. Here, we proposed that mechanical stretch could induce intracellular ROS production and further expression of NLRP3/pro-inflammatory cytokines in AF cells.

NLRP3 inflammasome is a key component of the innate immune system, and abnormal activation of the NLRP3 inflammasome has been implicated in various inflammatory diseases, including Alzheimer’s disease, diabetes, atherosclerosis, acute pancreatitis, fibromyalgia, etc. [24,25,26,27,28,29]. The activation of the NLRP3 inflammasome can be triggered by multiple molecular and cellular events, such as the production of ROS and mitochondrial dysfunction [27]. Moreover, the NLRP3 inflammasome mediates caspase-1 activation and secretion of the proinflammatory cytokines IL-1β/IL-18 in response to cellular injury, so the NLRP3 inflammasome has been indicated as an upstream regulator of IL-1β [27]. In addition, Liao et al. indicated that suppressing NLRP3 activation reduced synovial inflammation and fibrosis in knee osteoarthritis through the downregulation of IL-1β and IL-18 expression [30]. Recent studies, focusing on the mechanism of IVD degeneration, have demonstrated that activation of the NLRP3 inflammasome can result in apoptosis, inflammation and extracellular matrix (ECM) degradation in IVD tissue [31]. Similarly, ROS overproduction after IVD injury could cause a vicious circle of disc degeneration [32]. Previous studies have demonstrated that ROS can activate NLRP3 inflammasomes and then cause cellular damage by inducing caspase-1 activation and IL-1β secretion in the development of atherosclerosis, and IL-1β has been noted as the most important pro-inflammatory cytokine in IVD degeneration [33,34,35]. In the present study, we aim to determine the effects of the mechanical stretch on ER stress-related NLRP3/IL-1β expression in human AF cells (HAFCs). Therefore, different degrees and durations of cyclic stretch were applied to human AF cell lines to simulate daily spinal motion, in order to elucidate the effects of ER stress on NLRP3/IL-1β expression and ER stress-associated signaling pathways in AF cells under cyclic stretch.

## 2. Results

### 2.1. The 15% HCS Induced NLRP3 Expression in Human AF Cells

**Figure 1:**
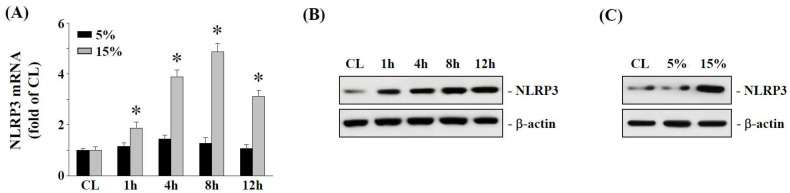
**HCS upregulates NLRP3 expression in human AF cells (HAFCs).** (**A**,**B**) HAFCs were exposed to 5% or 15% HCS for the indicated times, and the expression levels of NLRP3 mRNA (**A**) were determined by real-time PCR analysis. The results are mean ± standard error of the mean (SEM). The protein expressions of NLRP3 were determined by Western blot analysis (**B**). (**C**) HAFCs were kept as controls (CL) or exposed to 5% or 15% CS for 8 h, and the protein expressions of NLRP3 were determined by Western blot analysis. Bars in (**B**,**C**) are the mean and SEM fold change in band density relative to control, normalized to total protein levels from three independent experiments. * *p* < 0.05 versus CL.

The effect of the degree of cyclic stretching on NLRP3 mRNA expression was investigated in human AF cells using 5% light cyclic stretch (LCS) to simulate light tensile stress in the spine, whereas 15% high cyclic stretch (HCS) represented a high tensile stress condition. As shown in Figure 1A, 15% HCS induced NLRP3 mRNA expression in human AF cells as early as 1 h, reaching a maximum by 8 h and trending to decline at 12 h. The 15% HCS also increased NLRP3 protein expression of human AF cells in a time-dependent manner (Figure 1B). On the other hand, 5% LCS had less effect on NLRP3 gene and protein expression, suggesting that 5% LCS was insufficient to trigger inflammasome activation in AF cells for IVD degeneration (Figure 1A,C).

### 2.2. The 15% HCS Induced the Expression of IL-1β in Human AF Cells

**Figure 2:**
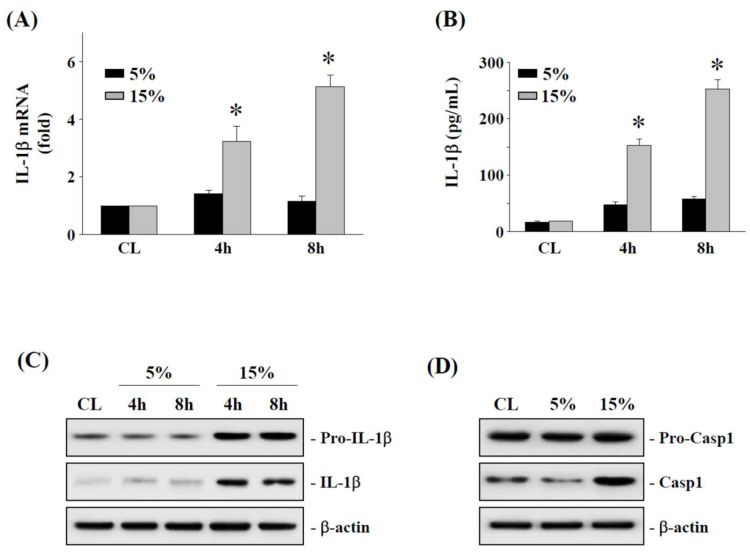
**Induction of IL-1β expression in HAFCs exposed to 15% HCS.** HAFCs were exposed to 5% or 15% cyclic stretch for the indicated times, and (**A**) the expression level of IL-1β mRNA were determined by real-time PCR analysis. (**B**) IL-1β secretion in medium was determined by ELISA analyses. The results are mean ± standard error of the mean (SEM). * *p* < 0.05 versus CL. (**C**,**D**) The expressions of pro-IL-1β and IL-1β (**C**), or pro-caspase-1 and caspase-1 (**D**) in HAFC lysate, were determined using Western blotting. Bars in (**C**,**D**) are the mean and SEM fold change in band density relative to control, normalized to total protein levels from three independent experiments.

To demonstrate that 15% HCS upregulated the expression and secretion of IL-1β, human AF cells were exposed to 15% HCS (relative to 5% LCS) at different time points (4 and 8 h). Time-course analysis determined that 15% HCS increased IL-1β mRNA expression at the 4 h and 8 h time points (Figure 2A). The 15% HCS also showed a similar pattern of increase in IL-1β secretion over the same time points (Figure 2B). In addition, the cytoplasmic pro-IL-1β and mature IL-1β protein levels were increased in human AF cells exposed to 15% HCS but not with LCS (Figure 2C). Following inflammasome activation, pro-caspase-1 underwent autocleavage to form caspase-1. Western blot analysis showed the increased protein level of caspase-1 (active form) at 4 h after 15% HCS (Figure 2D).

### 2.3. Effects of 15% HCS on NOX2 Expression and ROS Production in Human AF Cells

**Figure 3:**
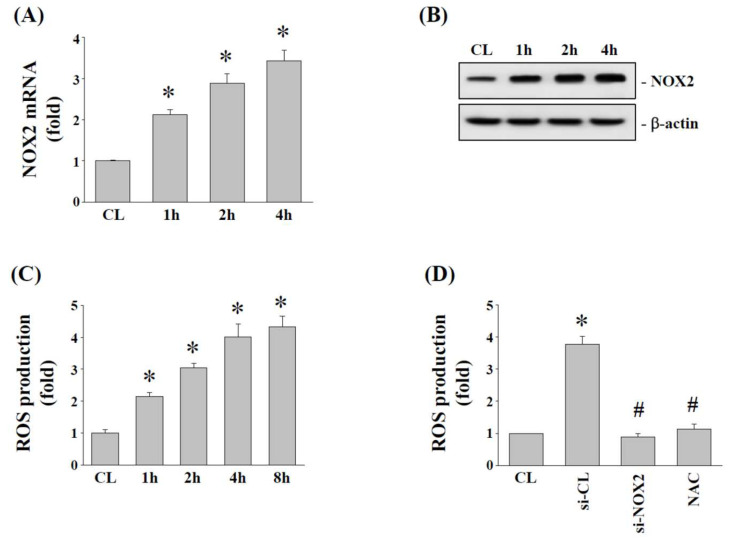
**HCSinduces NOX2 expression to trigger ROS generation.** (**A**,**B**) HAFCs were exposed to 15% HCS for the indicated times, and the expression levels of NOX2 mRNA (**A**) were determined by real-time PCR analysis. The protein expressions of NOX2 were determined by Western blot analysis (**B**). Bars in (**B**) are the mean and SEM fold change in band density relative to control, normalized to total protein levels from three independent experiments. (**C**) HAFCs were kept as the controls or were exposed to 15% HCS for the indicated times, and then the intracellular ROS level was examined. The results in (**A**,**C**) are mean ± standard error of the mean (SEM). * *p* < 0.05 versus CL. (**D**) HAFCs were kept as CL or exposed to 15% HCS. Before being kept as CL or exposed to stretch, HAFCs were transfected with control siRNA (si-CL), a specific siRNA of si-NOX2, or pretreated with NAC (ROS scavenger) for 1 h and then exposed to 15% HCS for 8 h, then the intracellular ROS level was examined. The results are shown as mean ± SEM. * *p* < 0.05 vs. CL. # *p* < 0.05 versus si-CL with 15% stretch stimulation.

NADPH (nicotinamide adenine dinucleotide phosphate) oxidases (NOX) and oxidative stress have been identified as important modulators for NLRP3 expression, and hence we determined whether 15% HCS affects NOX2 expression and ROS production in human AF cells. Cells were kept under static conditions as time-matched control or exposed to 15% HCS for the indicated times to examine NOX2 mRNA and protein expression. Compared with the control, 15% HCS significantly increased NOX2 mRNA (Figure 3A) and protein (Figure 3B) expression in human AF cells. Furthermore, ROS detection assay also confirmed that 15% HCS increased ROS production in human AF cells in a time-dependent manner (Figure 3C). To assess the role of NOX2 in modulating ROS production, human AF cells were transfected with control siRNA, NOX2 siRNA, or ROS inhibitor N-acetyl-L-cysteine (NAC, 0.5 mM), and then exposed to 15% HCS for 4 h. Cells transfected with NOX2 siRNA or pretreated with NAC significantly blocked 15% HCS-induced ROS production in human AF cells (Figure 3D).

### 2.4. ER Stress Regulates NOX2 Expression in Human AF Cells under 15% HCS

**Figure 4:**
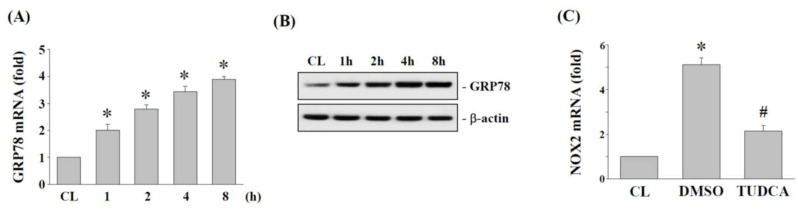
**HCS-induced NOX2 is mediated by ER stress.** (**A**,**B**) HAFCs were exposed to 15% HCS for the indicated times, and the expression level of GRP78 mRNA (A) were determined by real-time PCR analysis. The results are mean ± standard error of the mean (SEM). The protein expressions of GRP78 were determined by Western blot analysis (**B**). Bars in (**B**) are the mean and SEM fold change in band density relative to control, normalized to total protein levels from three independent experiments. * *p* < 0.05 versus CL. (**C**) HAFCs were kept as CL or exposed to 15% HCS. Before being kept as CL or exposed to stretch, HAFCs were pretreated with TUDCA (ER stress inhibitor) for 1 h and then exposed to 15% HCS for 4h, and the expression levels of NOX2 mRNA were determined by real-time PCR analysis. The results are shown as mean ± SEM. * *p* < 0.05 vs. CL. # *p* < 0.05 versus si-CL with 15% stretch stimulation.

ER stress is an important upstream activator for cellular oxidative stress. To determine whether 15% HCS regulates ER stress to induce NOX2 expression in human AF cells, cells were subjected to 15% HCS at different time points (1, 2, 4 and 8 h). Compared to the control, 15% HCS increased the mRNA (Figure 4A) and protein (Figure 4B) expression of GRP78 in a time-dependent manner in human AF cells. We also pretreated human AF cells with DMSO or ER stress inhibitor, TUDCA (200 μM), and then subjected them to 15% HCS for 4 h to demonstrate whether ER stress affects the stretch-induced effect on NOX2 expression. As shown in Figure 4C, TUDCA significantly blocked NOX2 expression induced by 15% HCS in human AF cells.

### 2.5. TXNIP and NLRP3 Expression Is Regulated by NOX2 and ER Stress in Human AF Cells under 15% HCS

**Figure 5:**
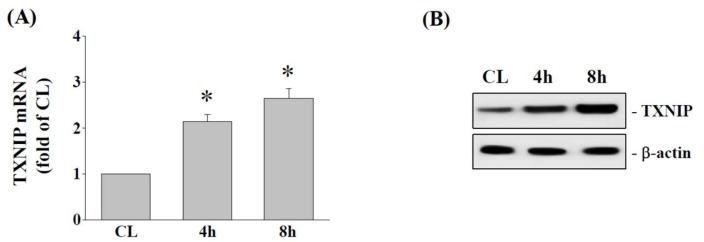
**HCS induces TXNIP expression in HAFCs.** HAFCs were exposed to 15% HCS for the indicated times, and the expression levels of (**A**) TXNIP mRNA were determined by real-time PCR analysis. The results are mean ± standard error of the mean (SEM). (**B**) The protein expressions of TXNIP were determined by Western blot analysis. Bars in (**B**) are the mean and SEM fold change in band density relative to control, normalized to total protein levels from three independent experiments. * *p* < 0.05 versus CL.

The association between TXNIP and NLRP3 plays an important role in NLRP3/IL-1β activation. To determine whether 15% HCS modulates TXNIP expression, cells were subjected to 15% HCS for 4 and 8 h, and we then determined TXNIP mRNA and protein expression levels. The results showed that 15% HCS significantly increased the expression of TXNIP mRNA (Figure 5A) and protein (Figure 5B) in human AF cells.

**Figure 6:**
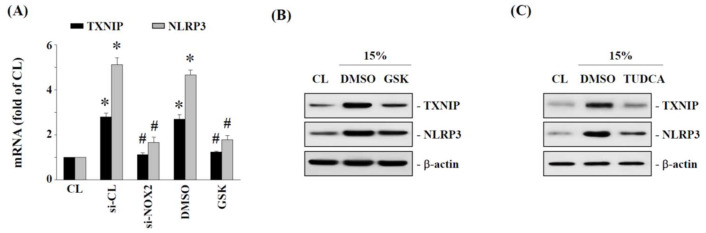
**Inhibition of ER stress and NOX2 decreasesHCS-induced TXNIP and NLRP3 expression.** HAFCs were kept as CL or exposed to 15% HCS. Before being kept as CL or exposed to stretch, HAFCs were transfected with control siRNA (si-CL), or a specific siRNA of si-NOX2, or were pretreated with DMSO, or specific inhibitors for NOX2 (GSK, 20 μM) or ER stress (TUDCA) for 1 h, and then exposed to 15% HCS for 8 h. (**A**) The mRNA expressions of TXNIP and NLRP3 were determined by real-time PCR. The results are shown as mean ± SEM. (**B**) The protein expressions of TXNIP and NLRP3 were determined by Western blot analysis. Bars in (**B**,**C**) are the mean and SEM fold change in band density relative to control, normalized to total protein levels from three independent experiments. * *p* < 0.05 vs. CL. # *p* < 0.05 versus si-CL or DMSO-treated cells with 15% HCS stimulation.

We further examined whether NOX2 and ER stress are involved in mediating 15% HCS-induced TXNIP-NLRP3 axis expression in human AF cells. Cells were transfected with NOX2-specific siRNA or the NOX2 inhibitor, GSK2795039 (20 μM), and then exposed to 15% HCS for 4 h to detect TXNIP and NLRP3 mRNA expression. Cells treated with si-NOX2 and GSK2795039 significantly blocked the mRNA expression of TXNIP and NLRP3 induced by 15% HCS for 8 h (Figure 6A). In addition, human AF cells were incubated with specific inhibitors of NOX2 (GSK2795039, 20 μM) and ER stress (TUDCA, 200 μM) for 1 h before and during exposure to 15% HCS. The results showed that GSK2795039 (Figure 6B) and TUDCA (Figure 6C) effectively suppressed 15% HCS-induced TXNIP and NLRP3 protein expression.

## 3. Discussion

IVD degeneration is considered to be a multifactorial disease [3,4,5,6,7,8,9,10], and the mechanism of IVD degeneration is not completely understood. Clinically, patients with degenerative spinal diseases are often those who perform labor, so heavy manual laborers have a higher incidence of degenerative spinal diseases due to frequent flexion and extension exercises, which could lead to excessive IVD load and accelerate IVD degeneration [6,7,10,12,36,37]. AF cells are thought to undergo tensile stress in vivo, and hence AF cells was designed to respond to cyclic stretch in a magnitude-dependent manner [22]. Mechanical loading with 15% HCS significantly increased the apoptosis of rat AF cells, and this was considered to be the physiological limit of stress observed in IVD [22,23,36]. Therefore, we chose a 15% HCS as the highest mechanical stress in this study. As expected, 15% HCS showed a significant increase in NLRP3 expression in human AF cells for each time point, but not with 5% LCS (Figure 1C), which suggested that 15% HCS may be important in the NLRP3 inflammasome activation in the pathogenesis of IVD degeneration.

Previous studies have reported that ER stress is a major underlying mechanism involved in various musculoskeletal system disorders, such as rheumatoid arthritis (RA) and osteoarthritis (OA), but how ER stress affects annulus fibrosus (AF) biology under mechanical stretch remains unclear [38]. GRP78 is an ER stress chaperone belonging to the heat shock protein 70 (HSP70) family, whose primary function is to regulate ER functions such as protein folding and aggregation and the breakdown of misfolded proteins [39,40]. Therefore, ER stress would increase the production of GRP78 [40]. In recent years, many studies have shown that excessive production of GRP78 can promote tumor cell proliferation, malignant metastasis, and increase drug resistance [39,40]. Here, we found that 15% HCS resulted in a significant time-dependent increase in GRP78 mRNA (Figure 4A) and protein (Figure 4B) expression in human AF cells. Thus, HCS could induce ER stress and promote the upregulation of GRP78 expression in AF cells.

Oxidative stress has been reported in a variety of diseases, including cardiovascular disease, lung injury, and central nervous system disease, etc. [41,42,43]. However, most research studying AF cells with tensile stress focus on the effects of cell apoptosis, inflammatory responses and ECM degradation to understand the progression of IVD degeneration, but the impact of tensile stress on oxidative stress has rarely been addressed in AF or NP cells [44,45]. Our results indicated that the gene and protein expression of NOX2 and ROS production were increased under 15% HCS stimulation (Figure 3A–C). Further analysis also showed that tensile stress upregulates the ROS/TXNIP pathway and promotes the expression of the inflammasome complex NLRP3 through ER stress in AF cells (Figure 5 and Figure 6). Consistent with our study, previous studies have demonstrated that oxidative stress would lead to excessive apoptosis and autophagy in IVD cells and further cause IVD degradation [15,33,46]. In addition, our data also demonstrated that the ER stress inhibitor, TUDCA, significantly inhibited 15% HCS-induced NOX2 expression (Figure 4C). Therefore, excessive tensile stress may increase ER stress by the expression of GRP78 and then promote ROS production through the upregulation of NOX2 expression in the progression of IVD degeneration.

Since the NLRP3 inflammasome is associated with a variety of inflammatory diseases [24,25,26,27], the relationship between NLRP3 and the mechanism of IVD degeneration is worth noting. Intracellular oxidative stress, mitochondrial dysfunction and ER stress, stimulated by internal or external factors, could activate the NLRP3 inflammasome in IVD cells [31]. Other signaling pathways, including the TXNIP/NLRP3/caspase-1 [47], Piezo1/NLRP3 [21], LINC00969/miR-335-3p/TXNIP [48], and cGAS/Sting/NLRP3 [49] axes, are also related to the regulation of the NLRP3 inflammasome activity and function [31]. Here, we further elucidated whether ER stress response is involved in the process of NLRP3 inflammasome activation in AF cells induced by HCS. Our data confirmed that 15% HCS could induce TXNIP expression and NLRP3 inflammasome activation, and that this can be suppressed by si-NOX2 or the NOX2 inhibitor GSK2795039 (Figure 5 and Figure 6). Moreover, the NOX2 inhibitor GSK2795039 and ER stress inhibitor TUDCA suppressed the protein expressions of TXNIP and NLRP3 in AF cells under 15% HCS.

Interleukin-1β (IL-1β), mainly regulated by the inflammatory complex NLRP3 [27], is highly expressed in DDD and is the major pro-inflammatory cytokine involved in the development of IVD degeneration [33,34,35]. NLRP3 activates and drives caspase-1 during the inflammatory response, resulting in the enzymatic hydrolysis of pro-IL-1β to form mature IL-1β and release [27]. The expression level of IL-1β has been proven to be positively correlated with the degree of IVD degeneration [35,50,51,52]. Furthermore, Chen et al. found a significant correlation between the severity of human disc degeneration and the mRNA and protein expression levels of NLRP3, caspase-1, and IL-1β [53]. Consistent with these findings, we found that high cycle stretch can regulate the NLRP3/IL-1β expression in AF cells through excessive production of GRP78 and caspase-1 at both mRNA and protein levels (Figure 2 and Figure 4). Therefore, activation of the NLRP3 inflammasome leads to overproduction of downstream IL-1β, which is implicated in the pathogenesis of human IVD degeneration, and our findings echo the possible role that NLRP3/IL-1β may play in IVD degeneration [53].

**Figure 7:**
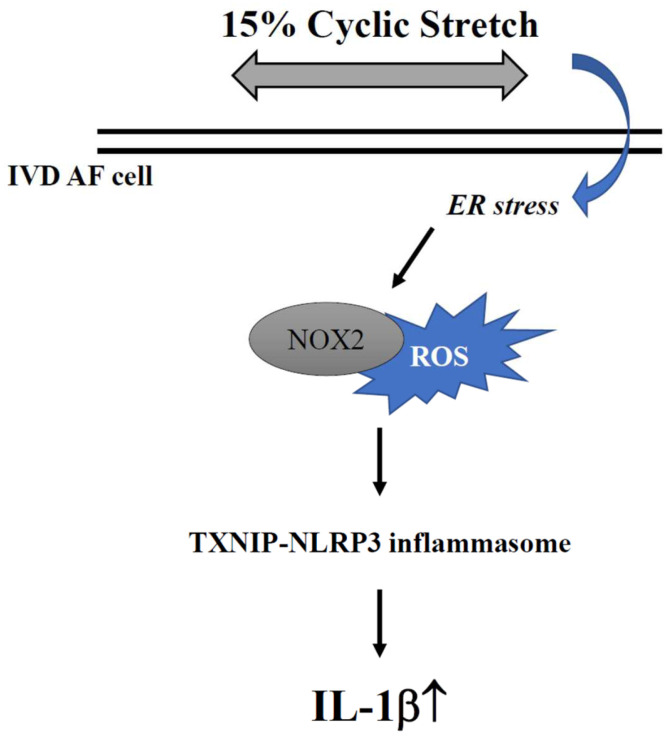
Schematic representation of a possible mechanism affecting 15% HCS-induced ER stress and NOX2 expression and consequent inflammasome activation in human AF cells.

In summary, we confirmed that HCS can induce ROS production and the consequent expression of NLRP3, caspase-1 and IL-1β in AF cells, and that this response is indeed upregulated by ER stress. In addition, human AF cells transfected with NOX2-specific siRNA or the NOX2 inhibitor GSK2795039 could significantly inhibit TXNIP and NLRP3 mRNA and protein expression under 15% HCS stimulation. A proposed molecular mechanism for the impact of cyclic stretch-related ER stress and associated NLRP3/IL-1β response on AF cells is illustrated in Figure 7. Thus, HCS-induced NLRP3 inflammasome/IL-1β-related inflammation in AF cells is regulated by ER stress.

## 4. Materials and Methods

### 4.1. Reagents

All culture materials were purchased from Gibco (Grand Island, NY, USA).

### 4.2. Culture of AF Cell Lines

The human AF cells were purchased from ScienCell Research Laboratories (Carlsbad, CA, USA) and grown in complete growth medium supplemented with 10% FBS. Cells at passage 3–6 were tested. After reaching 80% confluence, the cells were trypsinized and seeded onto stretching chambers.

### 4.3. Cyclic Stretching

Commercial stretching chambers (10 cm^2^, STB-CH-10, STREX, Taiwan) were coated with 50 µg/mL fibronectin (FC010, Merck Millipore, Darmstadt, Germany) overnight at 37 °C. The human AF cells were seeded in the chambers (100,000 cells per chamber in 5 mL growth medium) and cultured for 72 h at 37 °C, 5% CO_2_, unless otherwise stated. The cells were serum-starved for 12 h in a serum-free medium. The chambers were mounted on a commercial stretching bioreactor (STB-140-10, STREX, Taiwan) and subjected to 5% (light cyclic stretch, LCS) or 15% (high cyclic stretch, HCS) cyclic sinusoidal uniaxial strain at a frequency of 1 Hz at 37 °C and 5% CO_2_. Control chambers were kept in identical conditions without stretching. Cells were stretched for different amounts of time. The stretching started with the chambers that were stretched for 24 h, and shorter duration chambers were added progressively on the device so that the stretching was stopped at the same time for all conditions. Immediately after the mechanical loading, the cells were lysed for gene expression analysis.

### 4.4. Real-Time Quantitative PCR

Total RNA preparations and RT reactions were carried out as described previously [54]. Gene expression was analyzed by quantitative real-time PCR (ABI Prism 7900HT, CA, USA) using the SYBR Green PCR Master Mix (Applied Biosystems, Waltham, MA, USA). PCR products were subjected to melting curve analysis, and the data were analyzed by the 2^−ΔΔCT^ calculation method and standardized by GAPDH. The specific sequences of NLRP3 primer (plus-GAT CTT CGC TGC GAT CAA CA and minus-GGG ATT CGA AAC ACG TGC ATTA), IL-1β primer (plus-AAA CAG ATG AAG TGC TCC TTC CAG G and minus-TGG AGA ACA CCA CTT GTT GCT CCA), NOX2 primer (plus-CAA GAT GCG TGG AAA CTA CCT AAG AT and minus-TCC CTG CTC CCA CTA ACA TCA), GRP78 primer (plus-TGG GTC GAC TCG AAT TCC AAA G and minus-GTC AGG CGA TTC TGG TCA TTG G), TXNIP primer (plus-ACT CGT GTC AAA GCC GTT AGG and minus-TCC CTG CAT CCA AAG CAC TT), and GAPDH primer (plus-AGG TGA AGG TCG GAG TCA AC and minus-CCA TGT AGT TGA GGT CAA TG AAGG) were used in the real-time PCR.

### 4.5. IL-1β Enzyme-Linked Immunosorbent Assay (ELISA)

The levels of IL-1β in the media were determined by using sandwich ELISA kit (sensitivity 18 pg/mL) according to the manufacturer’s protocols (R & D Systems, Minneapolis, MN, USA)

### 4.6. Western Blot Analysis

Cells were lysed with a buffer containing 1% NP-40, 0.5% sodium deoxycholate, 0.1% SDS, and a protease inhibitor mixture (containing PMSF, aprotinin, and sodium orthovanadate). The total cell lysate (50 μg of protein) was separated by SDS-polyacrylamide gel electrophoresis (PAGE) (12% running, 4% stacking) and analyzed using the designated antibodies and the Western-Light chemiluminescent detection system (Bio-Rad, Hercules, CA, USA), as previously described [54].

### 4.7. siRNA Transfection

For siRNA transfection, cells were transfected with the designated construct using a RNAiMAX transfection kit (Invitrogen, Carlsbad, CA, USA). NLRP3-siRNA transfections caused at least an 80% reduction in the respective protein expression levels compared with the siRNA control vector (data not shown).

### 4.8. Statistical Analysis

The results shown in this study are expressed as mean ± standard error of the mean (SEM). Statistical analysis was performed using an independent Student t-test for two groups of data and analysis of variance (ANOVA) followed by Scheffe’s test for multiple comparisons. *p* values of less than 0.05 were considered significant.

## 5. Conclusions

HCS can induce ROS production, resulting in the expression of NLRP3 and IL-1β, and this response is indeed upregulated by ER stress. HCS-induced production of ROS and the consequent expression of NLRP3 and IL-1β are dependent on the upregulation of ER stress. HCS may accelerate the activation of the NLRP3 inflammasome complex in AF cells. This study focuses on the impact of HCS-related ER stress and associated NLRP3/IL-1beta response on AF cells. A better understanding of the regulatory mechanisms between HCS-induced ER stress and its NLRP3 inflammasome-related response on AF cells may help us develop strategies to reduce ROS levels in AF cells, thereby preventing the development of disc degeneration.

## Data Availability

The data that support the findings of this study are available until 5 years after publication to researchers who provide a sound proposal and all study sites and the Sponsor agree to sharing the data. Proposals should be directed towards the corresponding author.
